# Mitochondrial behaviour throughout the lytic cycle of *Toxoplasma gondii*

**DOI:** 10.1038/srep42746

**Published:** 2017-02-16

**Authors:** Jana Ovciarikova, Leandro Lemgruber, Krista L. Stilger, William J. Sullivan, Lilach Sheiner

**Affiliations:** 1 Wellcome Centre for Molecular Parasitology, University of Glasgow, UK; 2 Department of Pharmacology and Toxicology, Indiana University School of Medicine, Indianapolis, Indiana 46202, USA; 3 Department of Microbiology and Immunology, Indiana University School of Medicine, Indianapolis, Indiana 46202, USA

## Abstract

Mitochondria distribution in cells controls cellular physiology in health and disease. Here we describe the mitochondrial morphology and positioning found in the different stages of the lytic cycle of the eukaryotic single-cell parasite *Toxoplasma gondii*. The lytic cycle, driven by the tachyzoite life stage, is responsible for acute toxoplasmosis. It is known that whilst inside a host cell the tachyzoite maintains its single mitochondrion at its periphery. We found that upon parasite transition from the host cell to the extracellular matrix, mitochondrion morphology radically changes, resulting in a reduction in peripheral proximity. This change is reversible upon return to the host, indicating that an active mechanism maintains the peripheral positioning found in the intracellular stages. Comparison between the two states by electron microscopy identified regions of coupling between the mitochondrion outer membrane and the parasite pellicle, whose features suggest the presence of membrane contact sites, and whose abundance changes during the transition between intra- and extra-cellular states. These novel observations pave the way for future research to identify molecular mechanisms involved in mitochondrial distribution in *Toxoplasma* and the consequences of these mitochondrion changes on parasite physiology.

The various functions of mitochondria are governed in part by positioning of the organelle at relevant cellular locations. The dynamic nature of mitochondrial behavior is indicated by the multiple morphologies seen in various cell types and under various growth conditions. For example, several mammalian cell-types show morphologically heterogeneous and unconnected mitochondria in a steady state^1^. On the other hand, mitochondria can assume a highly connected tubular structure, e.g. during the development of cardiomyocytes^2^; or a highly fragmented morphology as in the case of yeast sporulation^3^. In another example, the eukaryotic parasite *Trypanosoma brucei* presents a small, unbranched tube-like mitochondrion during the life-cycle stage when it is not involved in ATP production and a branched mitochondrion when the TCA cycle is active^4^,^5^,^6^. Little is known about morphological changes that may occur in the mitochondrion of the eukaryotic unicellular parasite *Toxoplasma gondii (T. gondii*). Examination of mitochondrial morphology in the rapidly replicating life stage (tachyzoite), when intracellular, shows a single mitochondrion which is predominantly found in a lasso shape that spans the parasite periphery^7^,^8^. This lasso shape is the main morphology described for the *Toxoplasma* mitochondrion in the current literature. Interestingly, during tachyzoite cell division the mother mitochondrion maintains close proximity to the mother-cell periphery and is excluded from the growing daughters until the late stages of cytokinesis^9^.

*T. gondii* must reside within a nucleated host cell to grow and divide. The tachyzoite stage will spend time in the extracellular environment after egress from a host cell, when it seeks a new host cell to invade, and thus continues its “lytic cycle”. Recent observations suggest that extracellular tachyzoites are also found free in the blood stream of infected mice^10^. The transition from intracellular to extracellular conditions is accompanied by drastic changes in ion concentration and nutrient availability. The ability to survive outside the host cell, move around and then invade a new host cell is critical to the *T. gondii* lifestyle and virulence.

As a first step to tease out a potential function-morphology correlation in the *T. gondii* mitochondrion, we looked for any morphological changes that can be observed during the lytic cycle. We found that extracellular parasites exhibit drastic changes in mitochondrial morphology immediately after being released from the host cells. These changes are characterized by detachment of the mitochondrial tubule from the parasite periphery and its accumulation in concentrated regions in the cell. These changes seem directional and reversible upon host cell re-entry. Electron microscopy links these striking morphological dynamics to a change in the abundance of long patches of high proximity between the parasite’s mitochondrion and the parasite’s alveoli-sacs at its pellicle. These observations pave the way for future studies of the molecular mechanisms controlling apicomplexan mitochondrial behavior and how it contributes to survival of parasites between intra- and extracellular states.

## Results

### Morphological changes in the mitochondrion of extracellular tachyzoites

**Figure 1:**
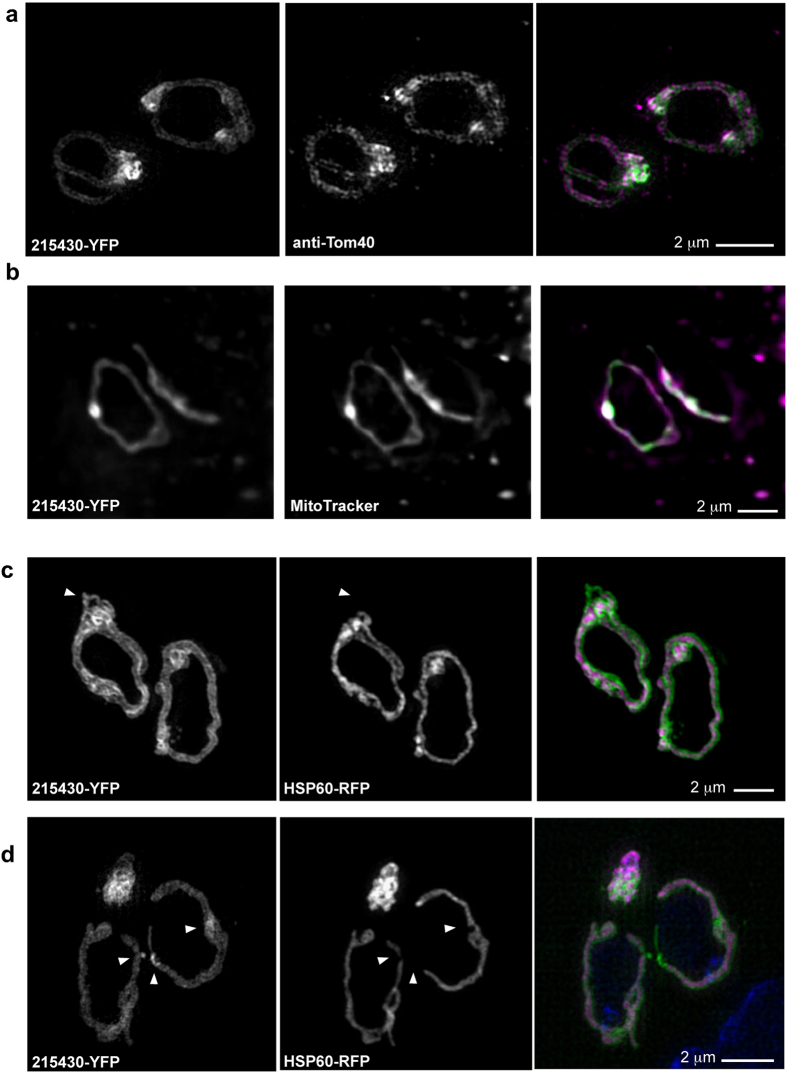
A new peripheral marker defines additional mitochondrial structures to a matrix marker. Immunofluorescence micrograph of the mitochondria of two intracellular *T. gondii* tachyzoite expressing 215430-YFP (green) and stained with anti-Tom40 antibody **(a)** or Mitotracker^®^ **(b).** All panels show Z projection. **(c,d)** Immunofluorescence micrograph of the mitochondria of two intracellular *T. gondii* tachyzoite expressing HSP60-RFP (magenta) and 215430-YFP (green). All panels show Z projection. Arrowhead in **(c)** highlight an example of 215430 marking additional structures to the matrix marker. Arrowheads in **(d)** point to places where the matrix signal break and the 215430 signal is continuous. Bars 2 μm.

Most previously available imaging of mitochondrial morphology and dynamics in live *T. gondii* utilized a matrix marker whereby the leader sequence of mitochondrial HSP60 is fused to the red fluorescent protein and the resulting fusion is expressed from a heterologous promoter^9^. We generated a fluorescent marker for the mitochondrial periphery via fusion of the TGME49_215430 encoded protein^11^ to the yellow fluorescent protein (YFP) by endogenous tagging. This protein was found in a search for *T. gondii* proteins that contain each a single hydrophobic domain, and within this screen it was localized to the mitochondrion (Sheiner and Soldati unpublished work). Homologs of this protein are only found in organisms from the Alveolata group (that includes within it the phylum Apicomplexa to which *T. gondii* belongs). No functional domains are predicted; however a lipid attachment site is predicted at the N-terminus (http://prosite.expasy.org/PS51257) that suggest potential attachment to the mitochondrial membrane. TGME49_215430 endogenously tagged with YFP (215430-YFP) co-localizes with the outer-mitochondrial membrane marker Tom40^12^ as well as the signal obtained from Mitotracker^®^ (Fig. 1a,b). We observed that imaging with this marker identifies mitochondrial structures that are not labeled using the matrix marker (Fig. 1c, arrowhead). It further labels a continuous mitochondrial tubule, whereas the matrix signal is fragmented (Fig. 1d, arrowheads). 215430-YFP is used throughout this report.

**Figure 2:**
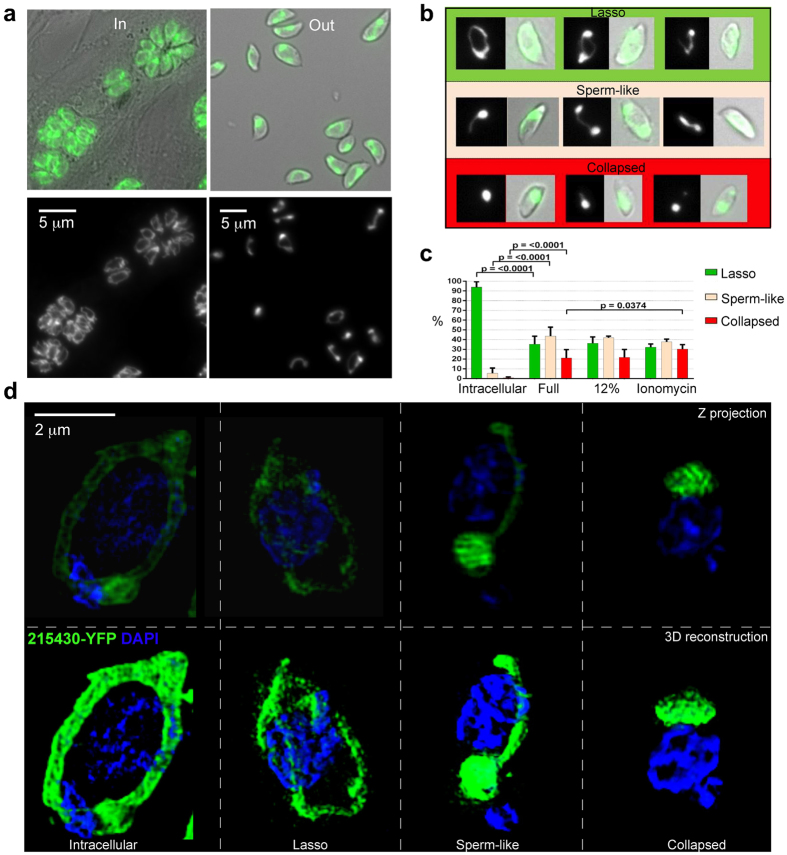
Mitochondrial morphology of *T. gondii* tachyzoites changes upon host cell egress. **(a)** Fluorescence micrograph of intracellular (In) and extracellular (out) populations of *T. gondii* tachyzoites taken utilizing the signal from 215430-YFP (green in the merge with the brightfield, top panels, and in the bottom panels). Bars 5 μm. **(b)** Three representative images of each of the observed shapes of mitochondria in extracellular parasites and their classification. Each example shows the fluorescent signal image on the left and the merge of fluorescence and brightfield on the right. The color-coding of the frame (lasso – green; sperm-like – orange; collapsed – red) is maintained throughout all the figures. **(c)** Proportions of the morphologies scored in intracellular parasites (Intracellular, 776 parasites, over 6 independent experiments); in parasites mechanically released from host cell into full growth medium (Full, 864 parasites, over 9 independent experiments) or into diluted growth medium (12%, 653 parasites, over 2 independent experiments) immediately after release; and in parasites induced to egress by 2 μM ionomycin (Ionomycin, 1177 parasites, over 6 independent experiments) immediately after release. Error bars are standard deviation. **(d)** Super-resolution microscopy images of the mitochondrial lasso morphology in intracellular (left) and the three main mitochondrial morphologies observed in extracellular: lasso, sperm-like and collapsed (right) shown as projection of all Z stacks (top) and as 3D reconstruction (bottom). 215430 - green. DAPI - blue. Bar 2 μm.

We revisited the previous observations on mitochondrial morphology in intracellular parasites. First we validated the new marker by reproducing the observations of the unique mitochondrial behavior in dividing tachyzoites^9^ (Fig. S1a–c, Movie S1). Next, we scored morphologies in intracellular parasites. *T. gondii* intracellular replication is asynchronous and different parasites are at different stages of the cell cycle at a given time. Nevertheless, we observed that an average of 94% of intracellular parasites possess the typical lasso shaped mitochondria with peripheral localization (Fig. 2), supporting earlier observations that this is the predominant morphology maintained throughout the intracellular cell cycle^7^. Live imaging show that the majority of parasites contain a lasso shaped mitochondrion also just before egress (Movie S2). However, due to the constant movement of the parasites within cells in the Z axis during imaging we were unable to determine the mitochondrial morphology with accuracy in all the parasites, making quantification difficult. In contrast, we found that immediately after host cell egress, *T. gondii* mitochondria present an array of morphologies (Fig. 2a,b), only 35% of which retain the lasso shape (Fig. 2c). We assigned these extracellular mitochondrial morphologies to three main categories based on their shapes and termed them “lasso”, “sperm-like” and “collapsed” (Fig. 2d). An average of 44% of freshly released parasites have “sperm-like” mitochondria (Fig. 2c), which consist of a round bundle of folded mitochondrial tubule, with a part of the tubule remaining extended. 21% of extracellular parasites show completely collapsed mitochondrial tubules (Fig. 2c). Similar distribution of these three morphologies is also observed when parasites egress into diluted medium that better mimics the low nutrient environment encountered by the parasite in the extracellular matrix *in vivo*^13^ (Fig. 2c). Equally, similar distribution of these morphologies is observed when parasites egress is induced via the calcium ionophore, ionomycin (Fig. 2c), with a moderate elevation in the proportion of collapsed mitochondria.

Changes in mitochondrial morphology were observed in *T. gondii* previously, in response to starvation and drug treatment. These studies describe mitochondrial swelling and fragmentation observed after 6 or 8 hours of treatment^14^,^15^. We noticed occasional freshly egressed parasites showing fragmentation or swelling similar to what was observed following starvation or drug treatment. In all cases, unlike the lasso, sperm-like and collapsed forms, this was coupled to an overall abnormal cell morphology documented by brightfield imaging (Fig. S2a). Comparison of these morphologies via super-resolution microscopy (3D-SIM) using the peripheral marker 215430-YFP showed that swollen and fragmented mitochondria have different structures to the packed folded tubule observed immediately upon host-cell release (Fig. S2b). Furthermore, extracellular parasites with swollen and fragmented mitochondria stain with propidium-iodide (PI), indicating cell death has commenced. Additionally, their mitochondria are not labelled with Mitotracker^®^, suggesting they are no longer active (Fig. S2c,d). Parasites with fragmented or swollen mitochondria were thus excluded from our analysis.

### Mitochondrion morphology during extracellular gliding motility, host cell invasion, and entry into a new host cell

**Figure 3:**
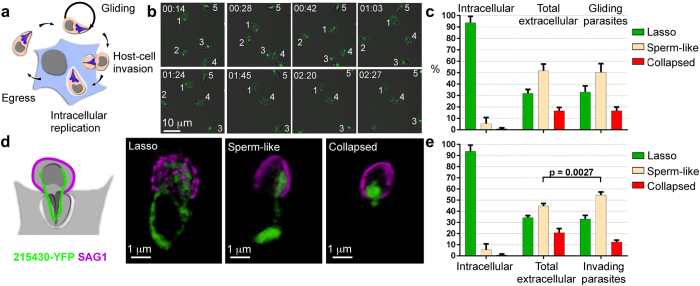
The change in the mitochondrial morphology of extracellular tachyzoites is seen in motile and in actively invading parasites. (**a**) A scheme depicting the main stages of the tachyzoite lytic cycle. (**b**) Snapshots from time-lapse microscopy of gliding parasites with different mitochondrial morphologies (Movie S3). Each parasite is numbered to enable following its trajectory. Time points (min:sec) are shown in each of the images. Bar −10 μm. (**c**) Distribution of lasso, sperm-like and collapsed morphologies in motile parasites (148 over 4 independent experiments), compared to the distribution in the total extracellular population (e.g. motile + non-motile, 171 parasites over the same 4 experiments), and to the distribution scored in intracellular parasites in the experiment presented in Fig. 2c. (**d**) Fluorescence images of invading parasites with lasso (left), sperm-like (middle) and collapsed (right) mitochondria. Snapshots from movies S4.1/2>/3. TGME49_ 215430 (green). SAG1 (magenta) staining was obtained without permeabilisation to visualize parasite that are half way into the host (i.e. only the extracellular part of the parasites is accessible to SAG1 antibody) as depicted in the scheme on the left. Bars −1 μm. (**e**) Distribution of mitochondrial morphologies in invading parasites (223 over 3 independent experiments) that were allowed to invade immediately after mechanical release from host cells, compared to the population of extracellular parasites (621 over 3 independent experiments) obtained from the same preparation of egressed parasites, and to the distribution scored in intracellular parasites in the experiment presented in Fig. 2c. Error bars in (**c,e**) are standard deviation.

During the lytic cycle (Fig. 3a), extracellular tachyzoites use gliding motility to find and enter their next host cell. We examined mitochondrial morphology in moving parasites upon temperature-shift induction of gliding motility in fresh mechanically-released parasites. The repertoire of morphologies in gliding parasites is similar to that of the overall population of freshly released parasites (33% lasso, 50% sperm-like and 17% collapsed) with no significant difference in the frequencies of each shape (Fig. 3b,c, Movie S3).

Host cell invasion is the next essential step of the *T. gondii* lytic cycle (Fig. 3a). Using the green/red assay (Ref. 16, Movie S4.1/2/3) to label invading parasites (Fig. 3d), we scored parasites that were in the act of invasion. We found a similar distribution of morphologies to those found in freshly egressed and in gliding parasites (33% lasso, 55% sperm-like and 12% collapsed), albeit a moderate increase in the frequency of sperm-like shaped mitochondria in the invading population (Fig. 3e).

**Figure 4:**
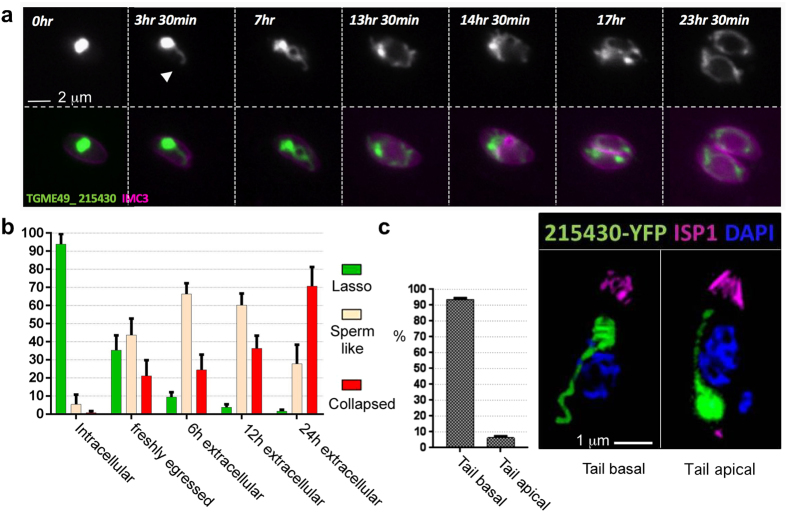
The observed morphological change is reversible upon host cell invasion and directional. **(a)** Snapshots from time-lapse microscopy of a parasite with collapsed mitochondrion after invasion and until completion of the first round of division (from Movie S5). 215430-YFP – green. IMC3 – magenta. Bar −2 μm. **(b)** Proportions of morphologies scored in parasites mechanically released from host cell after 6/12/24 hours of extracellular incubation (404, 930, 419 parasites were scored over 3,9,3 independent experiments respectively). These are compared to scores obtained immediately after release or in intracellular parasites from the experiment shown in Fg. 2c. Error bars are standard deviation. **(c)** Proportion of basal facing and apical facing “tail” in sperm-like mitochondria (190 parasites over 3 independent experiments, all counted after 6 hours of extracellular incubation, the time point with most sperm-like morphologies). The immunofluorescence on the right demonstrates examples of basal and apical facing tails. TGME49_ 215430 – green. ISP1 – magenta. DAPI - blue. Bar −1 μm.

Finally, parasites presenting the collapsed mitochondrial tubule morphology were analyzed after entry into a new host cell. Following invasion, collapsed mitochondria re-expand and re-establish the typical intracellular lasso shape (Fig. 4a, Movie S5). Among three independent live-imaging experiments the timing of re-expansion after invasion varied. However, cell division (as visualized by the appearance of daughter cells) occurred only after mitochondrial remodeling into a lasso shape (Fig. 4a, Movie S5). In this context it is worth mentioning that when examining cultures of intracellular parasites, the 0.7% that contain a collapsed mitochondrion (Fig. 2c) are always single parasites. We propose that these are parasites that recently invaded the host cell, and have not yet remodeled their mitochondrion to the lasso shape.

Interestingly, the transition from collapsed to lasso involves an intermediate sperm-like shape (Fig. 4a arrowhead). Due to the rapid motility of extracellular parasites we were unable to capture the remodeling in the opposite direction (from lasso to collapsed) via time-lapse microscopy of freshly egressed parasites. However, we found that parasites that remain extracellular for 0, 6, 12 and 24 hours, and that do not possess swollen and fragmented mitochondria, show 37%, 9.3%, 3.7% and 1.5% lasso-shaped mitochondria respectively (Fig. 4b). Within these time points the number of parasites with sperm-like mitochondria initially increased (from 45.5% to 66.2% at 6 hours) and then droped (to 27.8% at 24 hours), while parasites with collapsed mitochondria gradually accumulated to become 70.7% at 24 hours (Fig. 4b). This dynamic suggests a model whereby the sperm-like morphology is an intermediate form between lasso and collapsed. Analysis of the orientation of the tubule extension (“tail”) of the sperm-like mitochondria showed that the tail is predominantly basal (Fig. 4c), suggesting that the retraction from lasso to sperm-like is directional.

Collectively, these observations show that mitochondrial morphology in *T. gondii* tachyzoites is dynamic during the lytic cycle. The mitochondrion responds to the transitions between extracellular and intracellular stages in a controlled manner that results in the remodeling into a lasso shape observed after host cell invasion.

### Patches of tight mitochondrion-pellicle proximity are observed, and their abundance correlates with mitochondrial remodeling

We investigated the cellular structures linked to the peripheral positioning of the mitochondrion in intracellular tachyzoites. The behavior of mitochondria in other eukaryotic cells is controlled via several molecular mechanisms^17^. In many systems, components of the cell cytoskeleton play a central role in this process. We assessed mitochondrial morphology in response to treatment with the microtubule destabilization agent Oryzalin. Fixed cell microscopy showed that while this treatment results in irregular mitochondrion morphology, the results were distinct from the above described morphologies in extracellular parasites (Fig. S3a). Moreover, live-imaging of tachyzoite cell division under Oryzalin treatment shows that mitochondria enter the new daughter cells while still forming lasso-like shapes (Fig. S3b arrowheads, Movie S6).

**Figure 5:**
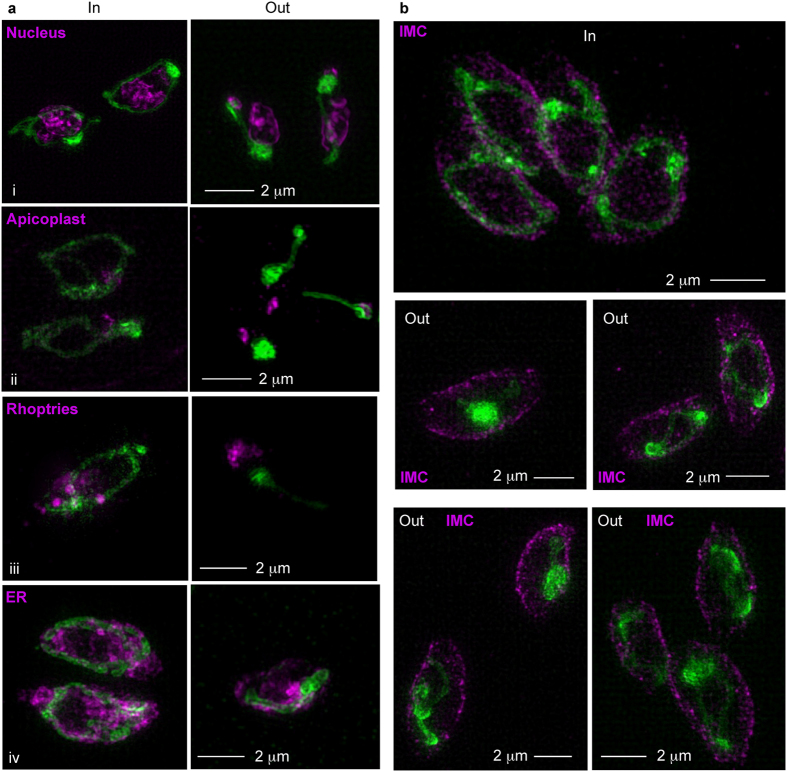
Analysis of morphology and positioning of other organelles in relation to the observed mitochondrial change reveals retraction from the parasite periphery as the main consistent feature of this change. (**a**) Randomly selected micrographs of the co-staining of mitochondrion and (i) nucleus (ii) apicoplast (iii) rhoptries (iv) ER in intracellular (in) and extracellular (out) parasites. (**b**) Representative micrographs of the co-staining of mitochondrion and IMC in intracellular (in) and extracellular (out) parasites. Bars −2 μm. TGME49_ 215430 - green. Anti-Rop4 antibody (Rop2,4 - T34A7^47^),/Der-GFP^48^/DAPI/anti-CPN60 - magenta.

Mitochondrial morphology is also shaped through its interactions with other organelles. We investigated whether other organelles show behavior that may be linked to the observed mitochondrial morphology changes upon the transition from intracellular to extracellular stages. We analyzed the morphologies and distribution of the three other organelles that occupy a large part of the tachyzoite cell and that show association with the mitochondrion in intracellular parasites^7^: the rhoptries, parasite-specific organelles that stretch from the apical tip to the nucleus; the nucleus; and the endoplasmic reticulum (ER) (Fig. 5a). No clear correlation between the positioning or morphology changes of these organelles and that of the mitochondrion was apparent (data not shown). We further examined the apicoplast, a relict plastid that shares metabolic pathways with the mitochondrion (reviewed e.g. in ref. 18), and for which an association with the mitochondrion is well-documented^9^,^19^. Specifically, we examined if the apicoplast is consistently found at a certain end of the sperm-like mitochondrion or at a certain end or distance from the collapsed mitochondrion. We further tested whether the apicoplast shape or location within the cell changes along with the mitochondrial shape-change. Again, no clear correlation was apparent (data not shown). However, imaging with the mitochondrial peripheral marker and a parasite pellicle marker (IMC3) revealed that all three extracellular morphologies show a trend of mitochondrial retraction from the tachyzoite periphery (Fig. 5b).

**Figure 6:**
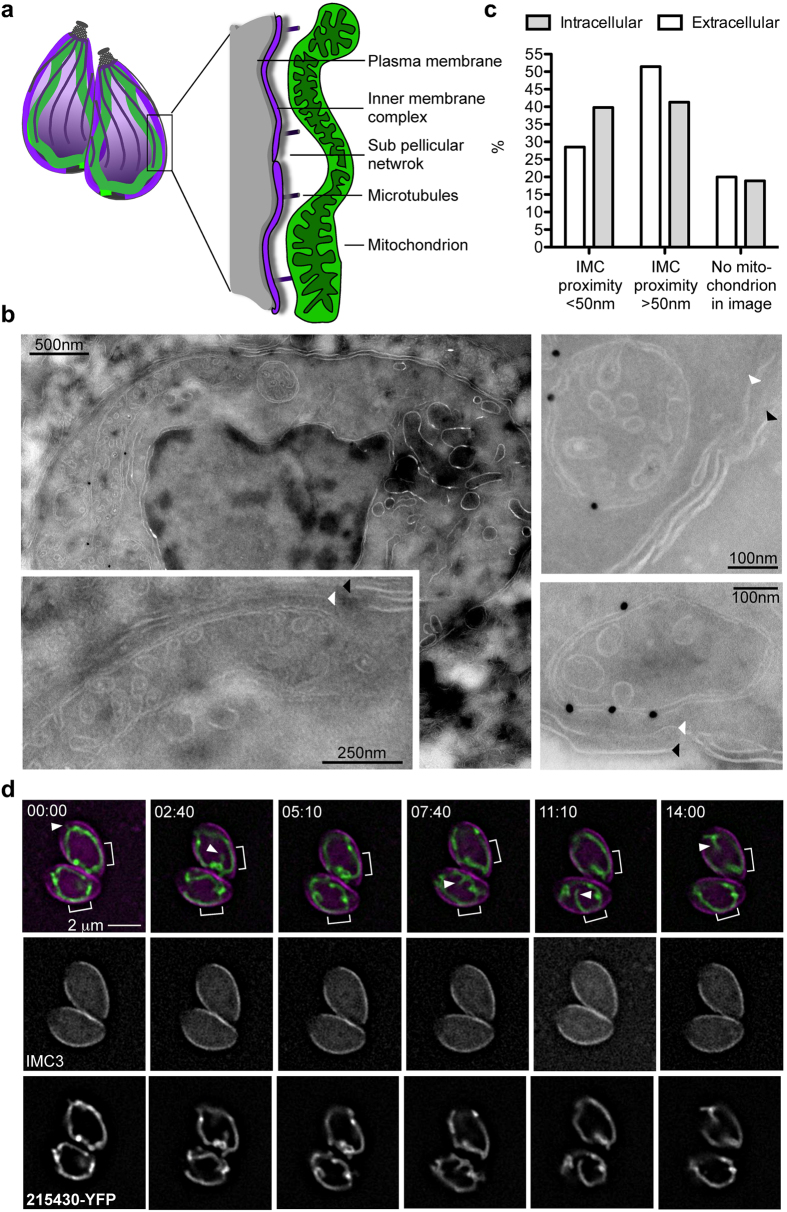
Mitochondrial peripheral retraction is linked to reduction in IMC tight-proximity zones. **(a)** Schematic depiction of two tachyzoites showing their mitochondria (green), plasma membrane (grey), microtubules (dark purple), inner membrane complex (magenta) and sub pellicular microtubules (fuzzy grey). **(b)** Cryo-immuno-EM using parasites expressing the mitochondrial associated TgElp3-HA^22^ (labeled with gold beads). White arrows show IMC. Black arrows show the plasma membrane. **(c)** Frequency of sections showing patches of mitochondrial–IMC proximity of <50 nm or >50 nm distance and of section not showing mitochondrial profile, among EM sections of intracellular and extracellular parasites. **(d)** Snapshots from the time-lapse microscopy shown in Movie S7. Merge panel shows TGME49_ 215430 in green and IMC3 in magenta, and each channel is also shown separately (IMC in the middle and TGME49_ 215430 at the bottom). Square parentheses highlight regions of close IMC-mitochondrion contact that are stable over the period of live imaging. Arrowheads mark region of transient extensions from the mitochondrial tubule.

The pellicle of *T. gondii* is multilayered (Fig. 6a Scheme); underneath the plasma membrane lays the inner membrane complex (IMC)^20^. The IMC is made up of flattened membrane sacs termed alveoli, covered, on the cytoplasmic face, by a network of intermediate filament-like proteins named the subpellicular network^21^. The IMC encircles the parasite periphery with openings only at the apical end (Fig. 6a Scheme) making it a strong candidate to interact with the mitochondrion and anchor its peripheral position in intracellular parasites. In support of this hypothesis, super-resolution microscopy showed substantial overlap in the signal from these two compartments (Fig. 5b, Fig. S1c arrowheads), which suggests proximity of less than 200 nm in the regions of overlap. Immuno-electron microscopy using cryofixation and labeling the outer mitochondrial membrane marker protein TgElp3^22^ (Fig. 6b), as well as electron tomography (Fig. S4), detected an abundance of regions of juxtaposition of mitochondrion and IMC, whereby the membranes of both organelles maintain constant distance (of less than 50 nm) over stretches of 100 nm–1000 nm. Among 254 random EM images of intracellular parasite sections, 39.8% presented the mitochondria within these patches of tight constant distance from the IMC; 41.3% presented more distant mitochondria; and 18.9% did not contain any mitochondria profile in the section (Fig. 6c). Within the images showing stretches of mitochondrion-IMC proximity of less than 50 nm, we measured an average distance of 26.23 nm (+/−12.02 nm). On the other hand, analysis of 240 EM images from freshly egressed extracellular parasites revealed 28.6% cases of mitochondrion-IMC alignment, while 51.4% showed no tight association and 20% showing no mitochondria profile in the section (Fig. 6c). The average distance between the IMC and mitochondria at the patches of alignment seen in extracellular parasites was 30.28 nm (+/−12.33 nm).

Finally, we tested whether the observed points of contact are stable over time by performing time-lapse microscopy with frequent imaging time points (every 10 seconds). We could image for periods of 10–30 minutes during which we observed contacts that lasted through the whole duration of the imaging (Fig. 6d, square parentheses, Movie S7) and up to 30 minutes. Occasional transient (no longer than 30 seconds) extensions towards the basal and apical end of the parasite as well as inward in the direction of the nucleus are also observed (Fig. 6d, arrowheads).

Collectively, these data show that the mitochondrion aligns closely with the IMC, and that this alignment is more extensive in intracellular parasites. We propose that this intimate association contribute to the mitochondrion shape and positioning seen in intracellular tachyzoites.

## Discussion

We describe new mitochondrial behavior in the protozoan parasite *T. gondii* whereby the transition between extracellular and intracellular stages induces changes in the organelle’s morphology. We identified three distinct morphological states that occur in extracellular tachyzoites and that are spotted immediately upon host cell egress; each state is observed with similar frequency in both the commonly used *in vitro* growth medium as well as a nutrient-reduced medium that more closely mimics the extracellular matrix *in vivo* (Fig. 2). Likewise, the distribution of these morphologies is seen in both moving and in actively invading parasites (Fig. 3). Finally, the change is reversible as collapsed mitochondria remodel into the typical lasso shape upon re-entry into the host. Taken together, these data suggest that the observed changes have physiological and functional significance.

Our observations point out a distinction between intracellular and extracellular mitochondrial morphology. The comparison between the two provided us with an opportunity to begin a dissection of the cellular features that may mediate the typical lasso shape of the mitochondria of intracellular tachyzoites. We did this by attempting to find a link between the behavior of certain cellular structures and the mitochondrial morphology. Mitochondrial morphology and localization in cells is controlled via interactions with components of the cytoskeleton and through interactions with other organelles^17^. Our analyses thus focused on both these factors.

Previous studies have shown that actin disruption results in morphological defects in mitochondria in both *T. gondii*^23^ and in the related parasite *Plasmodium falciparum*^24^. Likewise, treatments with microtubule stability inhibitors affect mitochondrial morphology (Ref. 9, Fig. S3). However, the peripheral distribution of the mitochondrion is still observed under both these treatments, and the three morphological states seen in extracellular parasite are not reproduced. Moreover, we recorded the formation of lasso-like shaped mitochondria when Oryzalin treated parasites divide (Fig. S3, Movie S6). We conclude that while components of the cytoskeleton appear to contribute to the control of mitochondrial morphology, additional factors are likely to be involved in mediating the peripheral positioning of the mitochondrion of intracellular tachyzoites.

Our analysis of the ER, nucleus, rhoptry and apicoplast in extracellular versus intracellular tachyzoites showed no consistent change in their cellular location or shape that coincided with the mitochondrial changes observed upon this transition (data not shown). This suggests that the interactions that exist between these organelles are not likely to contribute to the change of mitochondrial shape observed in our studies.

The predominant feature common to the three extracellular mitochondrial morphologies compared to intracellular mitochondria is a general retraction from the periphery (Fig. 5b), which correlates with a reduction in the number of patches where the mitochondrion is closely aligned to the IMC as observed by electron microscopy (Fig. 6). In other eukaryotes, regions of mitochondrial juxtaposition to other organelles are often attributed to the function of tether complexes that enable direct transmission of signals and molecules between organelles. These regions are named membrane contact sites (MCS) and have been reported to be present between any two organelles that have been closely studied^25^,^26^. The areas of apposition observed in this study between the mitochondrion and the IMC have similar length, shape and distance to those described for MCS, and they persist for 10–30 minutes at least (Movie S7, Fig. 6d). We therefore hypothesize that mitochondrion-IMC MCS are one of the factors responsible for the peripheral positioning and shape of mitochondria in intracellular tachyzoites, and that they are reduced upon the transition to the extracellular matrix. This interaction may also explain the previous observation showing that upon tubulin disruption mitochondria associate with local concentrations of IMC^9^. This hypothesis is also supported by findings from the closely related parasite *Plasmodium falciparum*, where, in sporozoites, long linker molecules that are apparently derived from the subpellicular network underlying the IMC, link the IMC with mitochondria^27^.

Some of the well-studied MCS involve mitochondria and include the ER–Mitochondria Encounter Structure (ERMES)^28^, the ER Membrane protein Complex (EMC) that also functions in ER-mitochondria tethering^29^, and the mitochondria and plasma-membrane tethering complex Num1p/Mdm36^30^,^31^. Mitochondrial contacts have also been observed with the yeast vacuole^32^ and peroxisomes^33^.

It is hypothesized that the IMC cisternae in Apicomplexa are of ER origin^34^ raising the possibility that complexes that tether the ER and mitochondria in other organisms may also tether the IMC and mitochondria in *T. gondii*. While no homologs of the ERMES complex components are identifiable in *Toxoplasma*, a set of EMC proteins homologs is present^35^. It has been postulated that in evolutionary lineages lacking ERMES, the more evolutionarily conserved EMC mediates the ER–mitochondrion tether. In humans, four other complexes were proposed to mediate mitochondrial-ER tethering (reviewed in ref. 26). Components of those complexes include the dynamin-like protein Mfn2, the mitochondrial fission mediator Fis1 and the mitochondrial voltage dependent anion channel (VDAC), all of which have homologs in *T. gondii* (Table S1) and are candidates to mediate these potential contact sites. Alternatively, a novel complex that is specific to these parasites or to the group of organisms containing alveoli, may tether the IMC and mitochondrion. Identifying tethers and confirming the existence of mitochondrial MCS in *Toxoplasma* is now a major priority in the field of organelle contact sites, that otherwise focuses mainly on opisthokonts, as it would expand to understand their role in divergent eukaryotes.

The possibility of mitochondrial-IMC communication raises the question of what functions are supported via exchange between these two organelles. The most commonly discussed functions of mitochondrial MCS are control of calcium homeostasis and trafficking of lipids. The lipid composition of the IMC and mitochondrion in *Toxoplasma* is not known. While lipid synthesis^36^,^37^ and lipid-dependent signalling pathways^38^ that are relevant to the function of both these organelles are being discovered, it is early to speculate on a potential role of mitochondrial-IMC contacts in lipid exchange.

Previous studies provide evidence both for and against a role for mitochondrion-IMC MCS in calcium homeostasis that may be similar to what is reported for mitochondrial-ER exchange in other systems. Regarding the mitochondria, *Toxoplasma* seems not to carry the gene encoding the mitochondrial calcium uniporter (MCU)^38^ that is involved in Ca^2+^ uptake in other systems. However, a mitochondrial antiporter which can mediate H^+^ -coupled Ca^2+^ exchange has a homolog in *Toxoplasma*^39^. As for the IMC, calcium storage in the *T. gondii* alveolar sacs has not been examined. However the alveoli of the related *Paramecium* spp act as calcium stores^40^, and this is also a suggested role of the IMC of the more closely related *Plasmodium* spp^41^.

MCS that mediate calcium homeostasis expand or reduce in respond to changes in calcium flux^42^. Treatments with ionomycin, which results in calcium flux in the parasite cytosol, induced a significant shift in the distribution of mitochondrial shapes (Fig. 2c) that may be a result of change in contact site length or abundance. Importantly, the observed shift is moderate. Moreover, neither depletion of lipids nor a modification of the overall medium composition to mimic the intracellular ion environment resulted in alteration to the mitochondrial shapes observed upon egress (Fig. S5). We cannot therefore conclude that there is any specific strong correlation between any single condition that we have analyzed and mitochondrial morphology. In this context, it is important to note that as observed in other eukaryotes, the activity of multiple tethers, executing various functions, may well contribute to the overall interaction between the same two organelles. Our hypothesis is that the observed mitochondrial change upon egress is the result of multiple changes encountered in this transition.

In addition to ions and lipids, other known roles of MCS that may have an effect here include the control of mitochondrial inheritance after cell division, and the regulation of the function of enzymes that work in trans (e.g. enzymes present on one organelle but modify substrates found on the other).

Identification of the molecular machinery responsible for the establishment of the patches of mitochondrion-IMC proximity would enable tackling its functional significance and test the above hypotheses. Likewise, understanding whether the peripheral retraction observed in extracellular parasites serves a function for extracellular survival, and/or it is the result of an intracellular function that is reduced upon egress, is an important step in the way of elucidating its role.

This work identified specific morphological states that occur to the mitochondrion in extracellular parasites, which are distinct from other stress-induced mitochondrial changes that have been previously described. These measures can now be used to assess the outcome of genetic ablation of potential mitochondrial biogenesis control mechanisms to address future questions designed to better understand why these remarkable changes in the mitochondrion take place.

## Methods

### Parasite culture

Parasites were grown in human foreskin fibroblasts (HFFs) in supplemented Dulbecco’s modified Eagle’s medium supplemented with 2mM L-Glutamine, with Pen/Strep antibiotic mix and 10% Fetal Bovine Serum (we refer to this as “full medium”). To generate fluorescent stable lines IMC3-YFP and TGME49_215430-tomato transgenes or an endogenous YFP tagging construct for TGME49_215430 were introduced into the RH based F3-line^43^, following enrichment of the fluorescent population of parasites stably expressing these transgenes via cell sorting using the S3e cell-sorter (BioRad). Clones were isolated by limiting dilutions.

### Immunofluorescence assay

All manipulations were carried out at room temperature. Intracellular parasites grown in HFFs seeded on glass coverslips were fixed with 4% paraformaldehyde for 20 minutes and washed in PBS. Extracellular parasites for immunofluorescence assay were placed onto poly-l-lysine coated coverslips and then fixed with 4% paraformaldehyde for 20 minutes and washed once with PBS. Cells were permeabilized and blocked in PBS/0.02% Triton-X-100/2% BSA (PBS/Triton/BSA) for 20 minutes. Slides were incubated for 60 minutes with primary antibodies: anti-TGME_215430^11^; ISP1^44^; IMC1^45^; Tom40^12^; and tubulin (Sigma) in PBS/Triton/BSA, washed with 3xPBS/0.02% Triton-X-100 and incubated for 45 minutes with Alexa488- or Alexa594-conjugated goat anti-mouse or anti-rabbit IgGs in PBS/Triton/FBS. For Mitotracker^®^ staining HFFs with parasites on glass cover slips were incubated in 300 nM Mitotracker^®^ for 30 min at 37 °C. For Oryzalin treatment intracellular parasites were cultured in presence of 2 microMolar Oryzalin for 18 hours after which the cells were fixed with 4% PFA for 20 minutes at RT and immunofluorescence assay was carried out as described above.

### Fluorescent Microscopy

Micrographs were obtained using DeltaVision Core microscope (AppliedPrecision) and processed using softWoRx and FIJI software. Parasites with heavily distorted cell shape and fragmented mitochondria were excluded from the analysis.

For super-resolution structural illumination microscopy (3D-SIM), stacks of 20–25 images were taken with increments of 0.091 μm in a Zeiss Elyra PS.1 super-resolution microscope (Jena, Germany) with a 63x oil immersion objective using ZEN Black software (Zeiss, Germany). Three-phase SR-SIM images were reconstructed in the same software using Structural Illumination manual processing tool. Maximum projection SR-SIM images and 3D models were processed in Zen and FIJI softwares^46^.

### Scoring Mitochondrial Morphology in Extracellular Parasites

215430-YFP expressing parasites were released from HFFs by needle pass (23 G, Henke Sass Wolf) and filtering through 3 μm filters (VWR, 515-2036). Parasites for immediate time point (Figs 2c and S5) were centrifuged (300RPM, 5 minutes) and fixed with 4% paraformaldehyde for 1 hour at room temperature. For egress in different medium compositions intracellular parasites are washed twice with the specific medium (12% full DMEM in Hanks-saline; FBS free medium; K^+^ buffer), parasites are then mechanically released by needle pass, filter, centrifuge and fixed as above. For chemical egress (Fig. 2c), intracellular parasites were incubated for 10 minutes in 2 μM ionomycin (Santa Cruz Biotechnology) in DMEM at 37° C prior to centrifugation and fixation. For the longer time points (Fig. 4b) scraped, needle passed and filtered parasites were incubated in 37° C, 5% CO_2_ in full medium for 6/12/24 hours before centrifugation and fixation. After fixation parasites were inoculated onto poly-l-lysine (Sigma) coated coverslips, allowed to adhere for 10 minutes at RT and washed once with PBS. Slides were mounted in DAPI Fluoromount-G^®^ (Cambridge Bioscience) and stored at 4 °C in the dark.

Morphologies were scored from micrographs obtained using DeltaVision Core microscope (Applied Precision) with x60 objective (example in Fig. 2a). Parasites looking small and round and with swollen or fragmented mitochondria were excluded from the analysis also in the longer incubation where their numbers were high. All error bars are standard deviation (mean with SD). The data for each mitochondrial shape was compared to the same mitochondrial shape in the control population using paired (gliding and invasion) or unpaired (egress method and media requirements) t-test. For the ionomycin experiment the control is Full DMEM; for gliding and for invasion the control is the total population counted from the same culture. For the 12% medium, FBS free medium and K^+^ buffer the control is full DMEM.

### Invasion assay

HFFs containing 215430-YFP expressing parasites where washed to remove extracellular parasites, and then scraped and needle pass (23 G) to release parasites. Freshly release extracellular parasites were used to infect new HFFs on glass coverslips. Cells were fixed with 4% paraformaldehyde after 30 minutes of incubation at 37° C. Following wash in PBS, cells were incubated in 2% BSA in PBS for 20 minutes. Cells were then incubated with anti-SAG1 antibody (Abcam) in 2% BSA in PBS for 1 hour and then with Alexa594 antibodies in 2% BSA in PBS for 45 minutes. The slides were mounted in DAPI Fluoromount-G^®^ (Cambridge Bioscience) and stored at 4 °C in the dark. Micrographs were obtained using 3D-SIM as detailed above.

### Gliding assay

HFFs containing 215430-YFP expressing parasites where washed to remove extracellular parasites, and then scraped and needle pass (23 G) to release parasites. Freshly release extracellular parasites were inoculated on pre-coated poly-l-lysine (1:10 in PBS) glass bottom dish (Cellvis) at 4 °C. The live dish was mounted on the imaging chamber of DeltaVision Core microscope (AppliedPrecision) preheated to 37° C. Images were taken every 7 seconds for a total of 10 minutes. Movie was compiled in FIJI software. For the movie used in this manuscript the images were processed to account for cell drifting using the ImageJ plugin, StackReg, for recursive alignment (http://bigwww.epfl.ch/thevenaz/stackreg/).

### Egress assay

HFFs containing 215430-YFP expressing parasites grown on glass bottom dishes (Cellvis) were washed to remove extracellular parasites. The dish was then mounted on the imaging chamber of DeltaVision Core microscope (AppliedPrecision) preheated to 37° C for live imaging. Images were taken every 10 seconds for a total of 5 minutes and 2 μM ionomycin (Santa Cruz Biotechnology) was added after 2–4 time points were imaged. Movie was compiled in FIJI software.

### Electron microscopy

RH strain *Toxoplasma* parasites expressing HA-tagged TgElp3 were prepared for immunoelectron microscopy (IEM) as previously described^22^. IEM processing and analysis was conducted by Wandy Beatty at Washington University, St. Louis. To immunolabel sections, a 1:25 dilution of rat anti-HA (Roche) was applied for 1 hour at room temperature. Samples were then incubated for another hour in a 1:30 dilution of goat anti-rat antibody conjugated to 18 nm colloidal gold (Jackson ImmunoResearch Laboratories) and stained with 5% uranyl acetate/2% methyl cellulose. Samples were analyzed on a JEOL 1200 EX transmission electron microscope (JEOL USA Inc.) with an AMT 8 megapixel digital camera and AMT version 602 software (Advanced Microscopy Techniques).

### Transmission electron microscopy

HFF infected cells and extracellular *T. gondii* tachyzoites were fixed with 2.5% glutaraldehyde and 4% paraformaldehyde in 0.1 M phosphate buffer. Following serial washes in 0.1 M phosphate buffer, the material was post-fixed in 1% OsO4 (vol:vol) in the same buffer for 1 hour on ice in the dark, and contrasted en bloc with 0.5% aqueous uranyl acetate for 1 hour at room temperature in the dark. The samples were then dehydrated in acetone ascending series and embedded in epoxy resin. Ultra-thin sections (60 nm) were observed in a Tecnai T20 transmission electron microscope (FEI, Netherlands). Images were processed and analyzed in FIJI software^46^. For the mitochondrion-IMC proximity analysis, 254 extracellular tachyzoites were imaged and 240 of infected HFFs. For the analysis, only distances less than 50 nm were considered as mitochondrion-IMC contact sites. The distances between IMC and mitochondrion profiles were measured in FIJI software, and data plotted in Microsft Exel software.

### Electron tomography

For 3D electron tomography, 200 nm-thick sections of infected HFFs were collected onto formvar-coated nickel grids. Images were recorded in tilt series covering +/−60°, at 2° increment intervals in a Jeol 2200 transmission electron microscope (Jeol, Japan) operating at 200 kV equipped with a Gatan US4000 camera. Tilt series were aligned by cross correlation and tomogram reconstruction calculated by weighted back projection using Etomo from IMOD software package (Kremer *et al*., 1996). Segmentation and generation of the 3D model were performed using 3dmod program of the same software package.

## Additional Information

**How to cite this article**: Ovciarikova, J. *et al*. Mitochondrial behaviour throughout the lytic cycle of *Toxoplasma gondii.* *Sci. Rep.* **7**, 42746; doi: 10.1038/srep42746 (2017).

**Publisher's note:** Springer Nature remains neutral with regard to jurisdictional claims in published maps and institutional affiliations.

## Supplementary Material

Supplementary Movie S1

Supplementary Movie S2

Supplementary Movie S3

Supplementary Movie S4.1

Supplementary Movie S4.2

Supplementary Movie S4.3

Supplementary Movie S5

Supplementary Movie S6

Supplementary Movie S7

Supplementary Information
