# Supplementary material for: Mitochondrial behaviour throughout the lytic cycle of Toxoplasma gondii
Source: Sci Rep. 2017 Feb 16;7:42746. doi: 10.1038/srep42746 (PMC5311943; doi:10.1038/srep42746)
Supplement: Supplementary Information [file srep42746-s10.pdf]

Mitochondrial behaviour throughout the lytic cycle of *Toxolasma gondii*

Jana Ovciarikova, Leandro Lemgruber, Krista Stilger, William J Sullivan Jr., and Lilach Sheiner

Supplementary materials and methods

Mitotracker® and Propidium Iodide staining

15 minutes prior to fixation, mitotracker® was added to the culture medium to a final concentration of 10 nM and left at 37° C. Parasites were inoculated onto poly-l-lysine coated coverslips, allowed to adhere for 10 minutes, washed once with PBS, fixed with 4% paraformaldehyde for 20 minutes and washed in PBS. The slides were mounted in DAPI Fluoromount-G® and stored at 4° C in the dark.

Propidium iodide was added to extracellular parasites at the final concentration of 1µg/mL. The parasites were moved to glass-bottom culture dishes (Cellvis) cover with poly-L lysin (Sigma, T9281) and imaged live using DeltaVision Core microscope (Applied Precision), 20 minutes after addition of propidium iodide.

Supplementary movies

Movie S1 – Two dividing parasites completing division from the moment of daughter IMC formation. IMC – magenta. 215430-YFP – green.

Movie S2 – Intracellular parasites induced to egress by the addition of 2µM ionomycin. The mitochondria are shown in the greyscale panel and in green in the merge.

Movie S3 – Freshly mechanically released parasites gliding after shift to 37° C, showing parasites with all three mitochondrial morphologies glide. 215430-YFP – green

Movie S4.1/2/3 – Rotating 3D reconstruction of an invading parasites stained with SAG1 (magenta) before premeabilization to label the parasite's extracellular part and then with anti-TGME49_215430 antibody (green) after permeabilization to label the mitochondrion. Each movie represents one of the observed extracellular morphologies: Lasso/Sperm-like/Collapsed in 3.1/2/3 respectively.

Movie S5 – A single recently invaded *T. gondii* with collapsed mitochondrion showing mitochondrial remodeling to an open peripherally proximal morphology followed by division. IMC3 – magenta. 215430-YFP – green

Movie S6 – Two dividing parasites under treatment with Oryzalin added at the start of imaging, completing division from the moment of daughter IMC formation. IMC – magenta. 215430-YFP – green division with oryzalin

Movie S7 – Two intracellular parasites at steady state, following mitochondrial morphology with time-lapse by imaging every 10 seconds. TGME49_ 215430 in green; IMC3 in magenta.

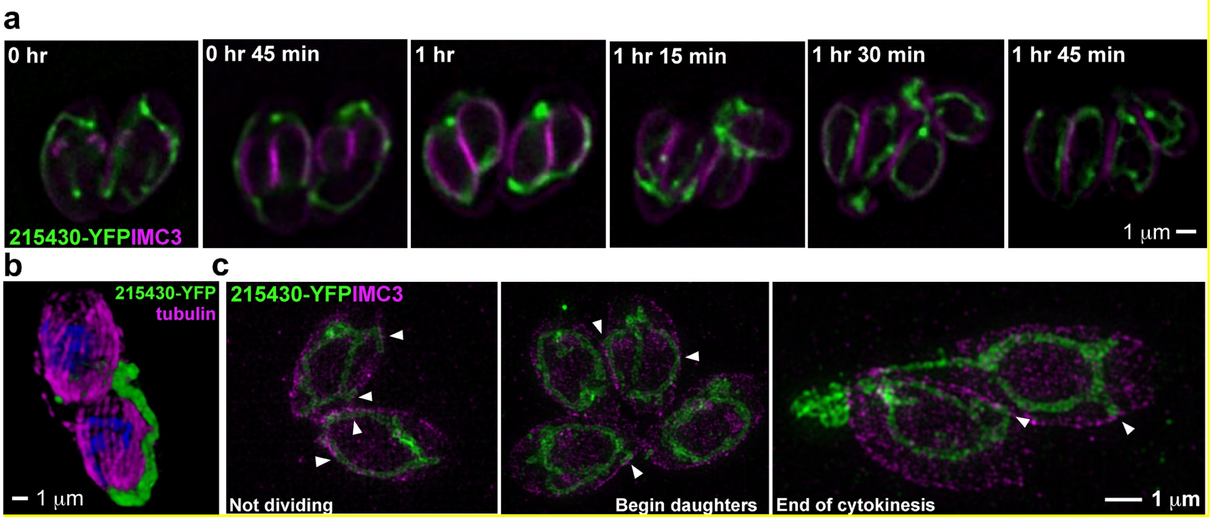

Figure S1. **A new mitochondrial outer membrane marker, 215430-YFP, recapitulates mitochondrial behavior in dividing tachyzoites and highlights peripheral proximity. (a)** Snapshots from time-laps microscopy (movie S1) showing mitochondrial behavior in two dividing parasites. 215430-YFP - green. IMC3 – magenta. Bar - 1μm **(b)** The typical mitochondrial exclusion from the growing daughter is shown by super resolution microscopy. Anti-215430 – green; anti-tubulin – magenta (mother tubulin signal is too weak to detect); DAPI – blue. Bar - 1μm. **(c)** Super-resolution microscopy showing mitochondrion (green) – IMC (magenta) overlapping signals at regions of mitochondrial extensions and tight aligning with the IMC (arrowheads) throughout the intracellular cell-cycle. Bar - 1μm

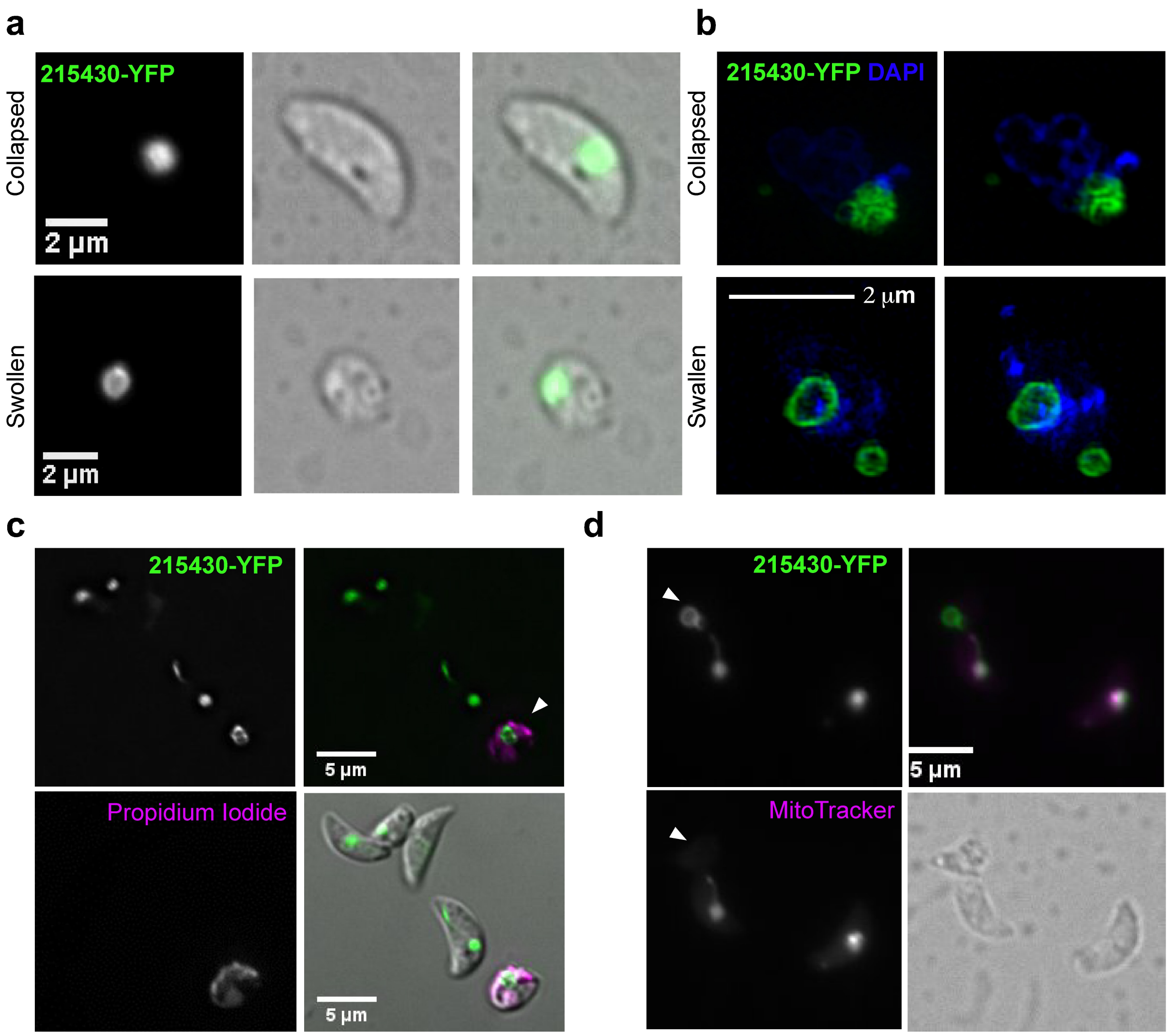

Figure S2. **Swollen/fragmented mitochondrial morphology is different to the herein observed collapsed mitochondria and occurs in dying cells**. **(a,b)** Collapsed (top panels) and swollen and/or fragmented (bottom panels) mitochondria (green) as documented by fluorescence (a) and super-resolution (b) microscopy shows different mitochondrial structures. DAPI – blue. Bars - 2µm. **(c)** Live/dead propidium iodide staining labels parasites with swollen and fragmented mitochondria (arrowheads) but does not label sperm-like or collapsed mitochondria. Bar - 5µm. **(d)** Mitotracker@ labels parasites with sperm-like or collapsed but not swollen (arrowheads) mitochondria. Bar - 5µm.

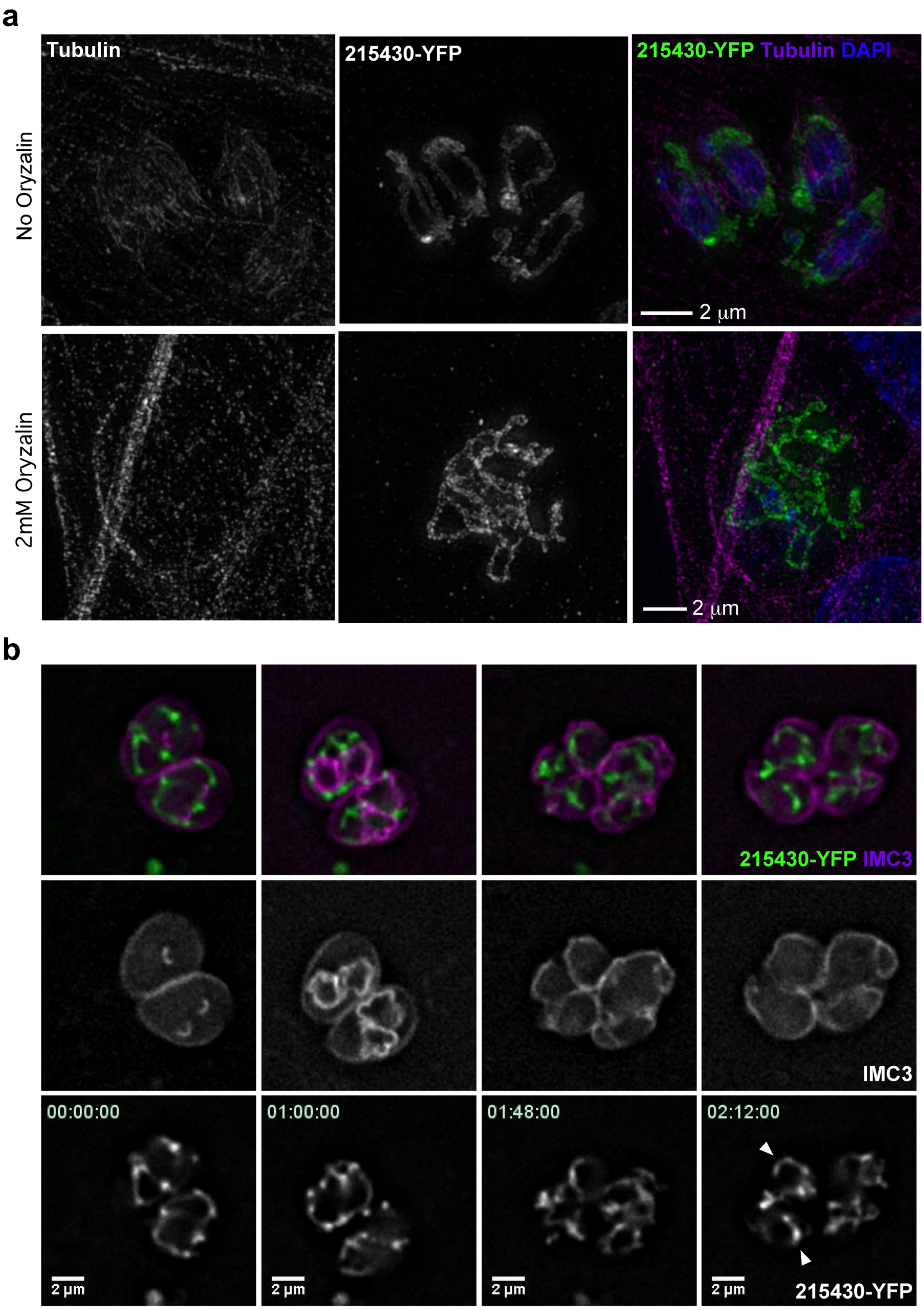

Figure S3. **Mitochondria peripheral proximity is maintained upon treatment with a microtubule disrupting agent. (a)** high resolution microscopy of parasites grown in absence (top) or presence (bottom) of 2 mM Oryzalin for 18 hours. Mitochondria - green. Microtubules - magenta. Bar - 2μm. **(b)** Snapshots from Time laps microscopy (movie S5) showing mitochondrial entry into the misshaped daughters formed under oryzalin treatment.

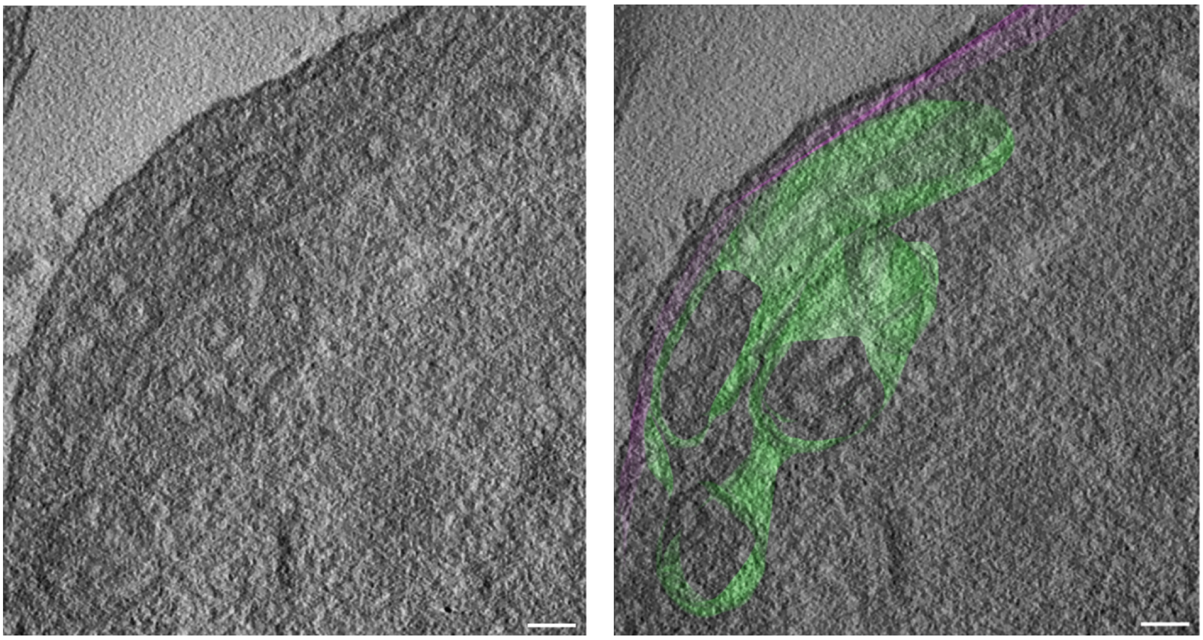

Figure S4. Electron tomography image of a representative section with a mitochondrion (green) – IMC (magenta) proximity patch.

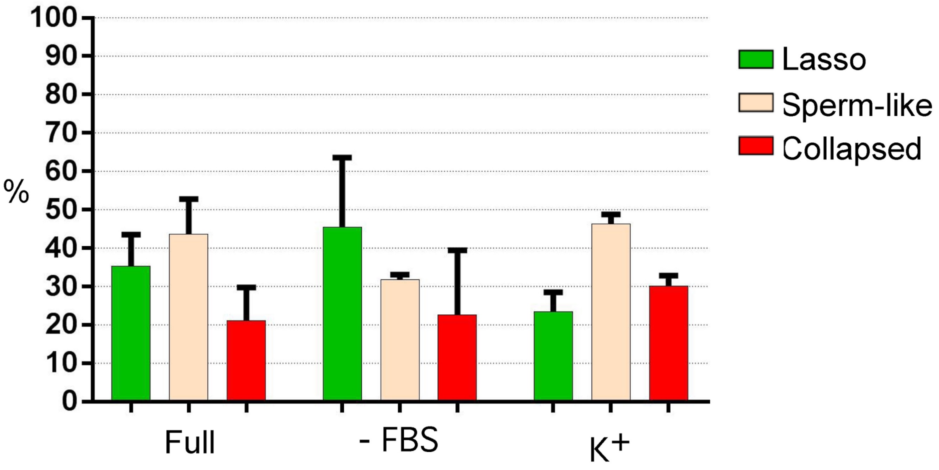

Figure S5. **Change in the composition of medium into which parasites are egressed have no significant effect on the distribution of mitochondrial shapes**. Proportions of the morphologies scored parasites egressed into medium with no FBS (- FBS, 252 parasites over 2 independent experiments) or into high potassium buffer (140 mM, NaCl 10 mM, MgSO4 2.7 mM, ATP (sodium salt) 2 mM, glucose 1 mM, EGTA 200 M, CaCl2 65 M (90 nM free Ca^2), and 10 mM Tris/Hepes, pH 7.3, K^+) (191 parasites scored over 2 independent experiments). These are compared to intracellular parasites and parasites mechanically released from host cell into full growth medium data from the same experiments shown in Figure 2c. Error bars are standard deviation.

Complex	Components	*T. gondii* homologs
ER–Mitochondria Encounter Structure (ERMES)	Mmm1	
	Mmm2	
	Mdm12	
	Mdm10	
ER Membrane protein Complex (EMC)	Emc1	TGME49_205740
	Emc2	TGME49_267840
	Emc3	TGME49_230100
	Emc4	TGME49_259000
	Emc5	TGME49_293200
	Emc6	TGME49_239690
	Emc7	TGME49_243390
	Emc8	TGME49_249310
	Emc9	
	EMC10	
Mitofusin 2	Mfn2	TGME49_070690*
		TGME49_121620*
		TGME49_067800*
VDAC/IP3R	VDAC	TGME49_263300
	IP3R	
	grp75	TGME49_251780
		TGME49_111720**
		TGME49_273760**
		TGME49_219310
VAPB/PTPIP51	VAPB	TGME49_318160
	PTPIP51	
Fis1/Bap31	Fis1	TGME49_263323
	Bap31	

Table S1 – ***T. gondii* homologs of components of complexes involved in mediating ER-mitochondria contact sites in other eukaryotes.** Homologs were identified by BLAST of the protein sequences of components identified in[43] against transcripts in ToxoDB (http://toxodb.org/toxo/). EMCs homolog were reported previously[30].

*Mitofusin 2 have no direct homologs in ToxoDB based on BLAST. Instead the 3 *T. gondii* dynamin-like proteins that are published[44] are listed.

**Published data is available for TGME49_111720[45] and TGME49_273760[46].
